# Progressive Multi-Scale Perception Network for Non-Uniformly Blurred Underwater Image Restoration

**DOI:** 10.3390/s25175439

**Published:** 2025-09-02

**Authors:** Dechuan Kong, Yandi Zhang, Xiaohu Zhao, Yanyan Wang, Yanqiang Wang

**Affiliations:** 1 School of Artificial Intelligence, Henan Institute of Science and Technology, Xinxiang 453003, China; kdc@hist.edu.cn (D.K.); wang_yy@hist.edu.cn (Y.W.); 2 National and Local Joint Engineering Laboratory of Internet Application Technology on Mine, China University of Mining and Technology, Xuzhou 221116, China; 3 School of Information Science and Engineering, Shenyang University of Technology, Shenyang 110870, China; zhangyd331@163.com

**Keywords:** underwater image enhancement, underwater non-uniform blur, multi-scale perception, hybrid interaction attention

## Abstract

Underwater imaging is affected by spatially varying blur caused by water flow turbulence, light scattering, and camera motion, resulting in severe visual quality loss and diminished performance in downstream vision tasks. Although numerous underwater image enhancement methods have been proposed, the issue of addressing non-uniform blur under realistic underwater conditions remains largely underexplored. To bridge this gap, we propose PMSPNet, a Progressive Multi-Scale Perception Network, designed to handle underwater non-uniform blur. The network integrates a Hybrid Interaction Attention Module to enable precise modeling of feature ambiguity directions and regional disparities. In addition, a Progressive Motion-Aware Perception Branch is employed to capture spatial orientation variations in blurred regions, progressively refining the localization of blur-related features. A Progressive Feature Feedback Block is incorporated to enhance reconstruction quality by leveraging iterative feature feedback across scales. To facilitate robust evaluation, we construct the Non-uniform Underwater Blur Benchmark, which comprises diverse real-world blur patterns. Extensive experiments on multiple real-world underwater datasets demonstrate that PMSPNet consistently surpasses state-of-the-art methods, achieving on average 25.51 dB PSNR and an inference speed of 0.01 s, which provides high-quality visual perception and downstream application input from underwater sensors for underwater robots, marine ecological monitoring, and inspection tasks.

## 1. Introduction

**Figure 1:**
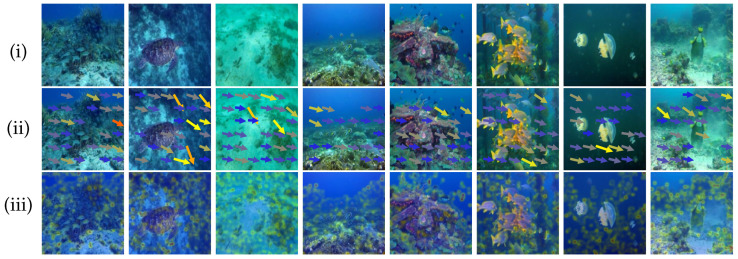
Illustration of non-uniform blur in underwater images. (**i**) The raw underwater images. (**ii**) The estimated blur trajectories, where the arrow directions and positions represent the motion blur vectors. (**iii**) The corresponding blur intensity heatmaps, where warmer colors indicate stronger blur magnitude.

With the advancement of marine science and technology, high-definition underwater imaging has become increasingly vital for underwater applications such as underwater robotic navigation, seabed topographic mapping, and aquatic life monitoring [1,2]. However, underwater imaging is affected by a variety of factors, including light absorption, scattering effects, and relative motion between the camera and the dynamic scene, resulting in image degradation, especially the non-uniform spatial blur distribution. We perform Fourier analysis on underwater images, extract the direction and intensity of localized frequency domain energy, and map them into blur direction and intensity heatmaps to visualize underwater blur patterns. As illustrated in Figure 1, real-world underwater images commonly exhibit non-uniform blur that varies in spatial extent and directional orientation, violating the common assumption of uniform blur kernels. Such degradation obscures critical visual cues, impairing the performance of downstream computer vision tasks. In autonomous underwater systems, the loss of structural information and motion cues due to non-uniform blur can lead to perceptual errors and decision-making biases, thereby posing challenges to the robustness and reliability of underwater operations.

To mitigate underwater image degradation, numerous hardware-based solutions have been developed, including specialized underwater cameras, structured lighting systems, and active imaging techniques such as laser illumination and time-gated imaging. These approaches aim to reduce visibility loss and scattering effects during image acquisition, thereby alleviating certain forms of blur. However, despite their potential to enhance raw image quality, such systems are commonly challenged for deployment in real-world environments due to their high cost, large physical footprint, and sensitivity to the environment. More critically, hardware-based methods do not explicitly address the underwater non-uniform blur arising from camera motion, moving objects, or dynamic water flow. This limitation has spurred increasing interest in algorithmic deblurring techniques, which can restore image sharpness directly from captured data without the need for auxiliary hardware.

Numerous studies have investigated traditional image enhancement techniques to address underwater image degradation. These methods typically leverage handcrafted priors and physical modeling to compensate for light attenuation and scattering. Common strategies include histogram equalization, white balance, gray-world assumptions, and Retinex-based methods for illumination decomposition. Some approaches further adapt atmospheric dehazing techniques, such as the Dark Channel Prior (DCP), to estimate the transmission map of underwater scenes. While these methods are relatively easy to deploy and interpretable, they generally assume static scenes and well-defined image structures, rendering them ineffective in the presence of the spatially non-uniform blur prevalent in real-world underwater environments. As a result, they commonly fail to recover fine image details and maintain structural consistency, limiting their utility in supporting high-level visual tasks.

With the growing application of deep learning (DL) in image restoration, learning-based methods for underwater image enhancement (UIE) have attracted increasing attention. Convolutional Neural Networks (CNNs) and Generative Adversarial Networks (GANs) are employed to correct color distortions, enhance contrast, and improve the visibility of underwater images, owing to their powerful feature extraction and representation learning capabilities. More recently, Transformer-based architectures have been introduced into underwater image enhancement and restoration tasks, demonstrating promising performance due to their global receptive field and long-range dependency modeling. However, most of these methods remain focused primarily on enhancing the visual quality of the image, lacking explicit mechanisms to model underwater image blur. Furthermore, the enhancement performance of the above networks often scales with their architectural complexity, which increases significantly in Transformer-based designs. This imposes substantial computational burdens, making real-time deployment in underwater applications impractical. Consequently, the performance of existing enhancement algorithms is frequently compromised under non-uniform blur conditions, underscoring the urgent need for dedicated deblurring frameworks tailored to the unique challenges of underwater environments.

Focusing on the challenge of non-uniform blur in underwater images, we propose PMSPNet, a Progressive Multi-Scale Perception Network. PMSPNet perceives and models blur features from multiple perspectives, encompassing both local and global receptive fields, contextual semantic information, as well as the direction and intensity of the blur. By integrating these diverse perceptual cues, the network effectively maximizes blur removal while enhancing overall image quality.

The main work of this article is summarized as follows:

- We propose a Progressive Multi-Scale Perception Network to effectively eliminate non-uniform blur in underwater images, enabling real-time underwater image enhancement.
- We introduce a Hybrid Interaction Attention Module that extracts and integrates local and global blur features to capture multi-view information and accurately perceive the direction and intensity of underwater blur.
- We design a Progressive Motion-Aware Perception Branch and a Progressive Feature Feedback Block to enable progressive fine-tuning of features, precise localization of blur, and efficient recovery of reconstruction details.
- We construct a Non-uniform Underwater Blur Dataset to provide a benchmark for evaluating underwater image deblurring algorithms. Extensive experiments demonstrate that the proposed method outperforms state-of-the-art approaches, validating its robustness and effectiveness.

The remainder of this article is organized as follows. Section 2 introduces related work. Section 3 describes the PMSPNet network. Section 4 presents the analysis and discussion of the experimental results, and Section 5 of this article concludes with a summary of the article and discusses potential future research areas.

## 2. Related Work

### 2.1. Hardware-Based Approach

To address the challenges of underwater image degradation, numerous hardware-based methods have been proposed. These approaches employ specialized imaging systems to capture higher-quality data at the point of acquisition, thereby reducing dependence on post-processing algorithms [3]. Some systems utilize auxiliary light sources or polarization filters to suppress scattering and backscatter effects [4,5]. In contrast, others adopt structured light or multi-camera setups to reconstruct clearer underwater scenes [6,7]. Notably, high-speed cameras and inertial measurement units (IMUs) have been leveraged to estimate and compensate for camera motion, thereby mitigating motion blur during image capture [8]. In parallel, the emergence of IoT-enabled sensor networks has driven research on adaptive routing, data reliability, and energy efficiency, which are critical for real-time underwater monitoring and communication systems [9,10,11]. These studies explore strategies such as cross-layer optimization, secure data aggregation, and energy-aware routing to improve the dependability and scalability of sensor-based infrastructures [12,13,14], providing complementary insights into the broader landscape of underwater sensing and communication. Integrating underwater image restoration techniques with efficient IoT-based sensing systems represents a promising direction, enabling both high-quality perception and reliable data delivery in challenging underwater environments. Although such hardware-enhanced systems can yield superior image quality under controlled conditions, they are commonly constrained by practical limitations, including high cost, limited deployment flexibility, and susceptibility to failure in dynamic or harsh underwater environments. Consequently, there is a pressing need for software-based solutions that can adaptively enhance underwater images without relying on specialized equipment, offering greater practicality, scalability, and robustness in real-world applications.

### 2.2. Traditional Approach

Traditional approaches to underwater image enhancement typically rely on physical priors or hand-crafted models to address degradation caused by absorption, scattering, and turbidity [15]. Zhou et al. employed a modified underwater image formation model incorporating depth estimation and color correction to effectively mitigate the effects of light absorption and scattering [16]. Ma et al. proposed an improved Retinex-based variational model that integrates information entropy smoothing and non-uniform illumination priors, enabling effective handling of uneven lighting in underwater images [17]. Liu et al. introduced an illumination-constrained, structure-preserving Retinex model with adaptive channel compensation and joint estimation of illumination and reflection, demonstrating competitive performance on turbid underwater images in both subjective and objective evaluations [18]. Zhou et al. combine pixel distribution remapping with a Retinex variational model and noise-texture priors, achieving notable improvements in color correction and contrast enhancement [19].

Beyond physics-based models, some methods focus on enhancing images by improving contrast, correcting color, or reducing haze based on prior knowledge or statistical assumptions without explicitly modeling the underwater imaging process. Zhang et al. proposed a principal component fusion method (PCFB) that enhances underwater images by fusing contrast-enhanced foregrounds and dehazed backgrounds using principal component analysis [20]. Zhang et al. introduced a multi-channel adaptive fusion approach that addresses color distortion and contrast loss through adaptive channel correction and dual-branch enhancement [21]. To accommodate images across different color gamuts, Zhang et al. proposed the RAG-IMF method, which integrates global–local color correction and multi-channel fusion in both RGB and LAB color spaces, thereby extending color gamut and improving quality metrics [22]. Similarly, Jha et al. developed the CBLA method, which performs RGB-based color correction and LAB-based contrast and naturalness restoration for effective underwater enhancement [23]. To preserve fine details, recent works have also explored multi-scale representations and frequency-domain techniques, leading to more robust enhancement across a variety of underwater scenes [24,25].

While the above approaches offer strong interpretability, their performance tends to degrade in highly dynamic underwater environments, where the underlying model assumptions are frequently violated. As a result, they are generally less effective in handling complex, non-uniform degradations such as motion blur and spatially varying illumination.

### 2.3. Data-Driven Approach

With the rapid advancement of deep learning, data-driven approaches have emerged as a powerful alternative for UIE [26]. Unlike traditional methods, data-driven techniques can implicitly model complex nonlinear degradations and adapt to diverse underwater conditions through training on large-scale datasets. Xue et al. addressed the limitations of conventional color spaces by introducing a learnable Underwater Scenes Orient (USO) color space and a Scene-Adapted Semantic-Aggregated Degradation-Decoupling (S2D2) framework [27]. Park et al. proposed a lightweight enhancement framework based on an adaptive standardization and normalization network, which effectively corrects distorted feature distributions and improves image contrast and brightness, all while maintaining low computational complexity [28].

In parallel, Generative Adversarial Networks (GANs) [29,30], particularly diffusion models [31,32], have further improved the capacity of neural networks to learn complex mappings between degraded underwater images and their high-quality counterparts. These models have demonstrated significant improvements in addressing color distortion, motion blur, and low contrast [33,34]. The recent success of Transformers in computer vision has further accelerated progress in underwater image enhancement [35,36]. Their powerful global modeling capabilities and context-aware mechanisms significantly improve detail restoration and color correction, resulting in more natural and visually appealing enhancement outcomes. Yang et al. introduced a progressive aggregation framework that utilizes a feature-prompted Transformer, combining global-local attention with multi-scale feature aggregation, to enhance detail preservation, color fidelity, and blur removal [37]. Huang et al. proposed an underwater enhancement network incorporating a cross-wise Transformer module and a feature supplementation strategy to capture inter-stage dependencies and compensate for feature loss [38]. Moreover, techniques involving multi-domain feature extraction, physics-guided priors, and unsupervised learning have been actively explored to improve the perceptual and generalization abilities of enhancement networks [39,40,41,42].

Despite their promising results, data-driven methods also face notable limitations. Convolutional Neural Networks (CNNs) struggle to capture long-range dependencies due to their localized and static receptive fields. Although Transformers effectively address this issue, their high computational cost limits their practicality, especially for deployment on resource-constrained edge devices. GAN-based models, while exhibiting strong feature generation capabilities, often suffer from limited generalization across diverse underwater scenes. Furthermore, the aforementioned methods require large volumes of training data, yet datasets specifically addressing underwater blur remain scarce. Therefore, the models are prone to domain bias, making them less effective in handling complex and variable non-uniform underwater blurred images.

## 3. Methods

**Figure 2:**
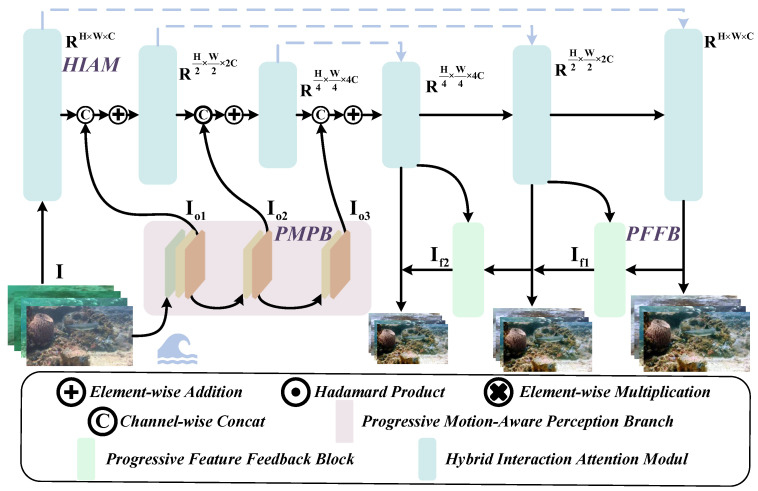
PMSPNet network architecture. Core components include the Hybrid Interaction Attention Module (HIAM), Progressive Motion-Aware Perception Branch (PMPB), and Progressive Feature Feedback Block (PFFB). The data come from the Non-uniform Underwater Blur Dataset (N2UD).

To address the challenges, we propose PMSPNet, a Progressive Multi-Scale Perception Network, as illustrated in Figure 2. The network incorporates a Hybrid Interaction Attention Module (HIAM) to capture local details and global contextual dependencies, enabling an initial coarse perception of image blur features. Building upon this, we introduce the Progressive Motion-Aware Perception Branch (PMAB) and the Progressive Feature Feedback Block (PFFB), which can incrementally guide and refine the network’s perceptual capability, thereby achieving accurate localization and representation of non-uniform blur regions. Furthermore, to facilitate more realistic and comprehensive training and evaluation, we construct a dedicated Non-uniform Underwater Blur Dataset (N2UD), which encompasses a wide range of blur patterns encountered in real-world underwater environments.

### 3.1. N2UD

**Figure 3:**
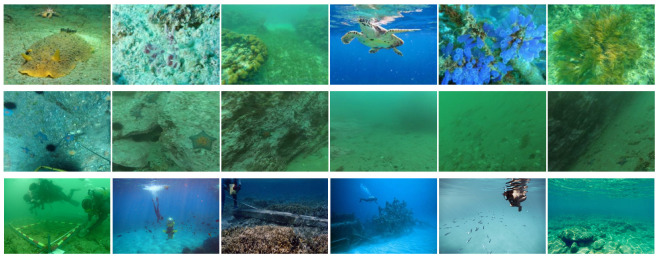
The N2UD dataset, which contains various underwater environments and different degrees of non-uniform blur.

To address the scarcity of underwater blur datasets, we constructed a specialized dataset by filtering an existing publicly available underwater image dataset. Specifically, we combined quantitative blur metrics with manual visual inspection to identify and select images exhibiting non-uniform blur. The resulting collection forms a new benchmark, termed the Non-uniform Underwater Blur Dataset (N2UD), which serves as a representative testbed for evaluating underwater deblurring algorithms under complex and realistic degradation conditions. As illustrated in Figure 3, N2UD includes underwater images affected by non-uniform blur across various resolutions and scenes, ensuring diversity and practical relevance.

The quantitative blur metrics of which can be expressed as

(1) $$\mathrm{isBlurred}=\left\{\begin{array}{cc} 1 & \delta_{U}+\delta_{C}+\delta_{G}+\delta_{H}>1\\ 0 & \mathrm{otherwise} \end{array}\right.,$$

where $\delta_{U}$ denotes the underwater image sharpness measure, $\delta_{C}$ represents the global contrast level, $\delta_{G}$ is the mean image gradient, and $\delta_{H}$ corresponds to the high-frequency energy. When IsBlurred equals 1, it signifies that the image exhibits non-uniform blurring. The underwater image sharpness measure $\delta_{U}$ is defined as

(2) $$\delta_{U}=\left\{\begin{array}{cc} 1 & \frac{1}{k_{1}k_{2}}\sum_{i=1}^{k_{1}}\sum_{j=1}^{k_{2}}20\cdot \log\left(\begin{array}{c} \frac{I_{\max}^{\left(ij\right)}}{I_{\min}^{\left(ij\right)}+\epsilon } \end{array}\right)<U_{t}\\ 0 & \mathrm{otherwise} \end{array}\right.,$$

where the image is converted to the YUV color space and then partitioned into $k_{1}\;\times \;k_{2}$ non-overlapping blocks; $I_{\max}^{\left(ij\right)}$ and $I_{\min}^{\left(ij\right)}$ represent the maximum and minimum luminance values, respectively, for the (i,j)th block; ϵ is a small constant ($10^{-6}$) to avoid division by zero; and $U_{t}$ is the corresponding threshold, set to 2.0. The $\delta_{C}$ is expressed as

(3) $$\delta_{C}=\left\{\begin{array}{cc} 1 & \frac{\sigma (I_{\mathrm{LAB}}^{\left(l\right)})}{255}<C_{t}\\ 0 & \mathrm{otherwise} \end{array}\right.,$$

where $I_{\mathrm{LAB}}^{\left(l\right)}$ is the LAB color space of the input image $I_{\mathrm{RGB}}$, σ(·) is the standard deviation, and $C_{t}$ is the corresponding threshold, set to 0.15. The $\delta_{G}$ is expressed as

(4) $$\delta_{G}=\left\{\begin{array}{cc} 1 & \frac{1}{N}\sum_{x=1}^{W}\sum_{y=1}^{H}M\left(x,y\right)<G_{t}\\ 0 & \mathrm{otherwise} \end{array}\right.,$$

where *N* is the total number of pixels in the image; W, H are the width and height of the image, respectively; and $G_{t}$ is the corresponding threshold, set to 15. The M(·) is the gradient magnitude calculation for each pixel point, which is expressed as

(5) $$M\left(x,y\right)=\sqrt{G_{x}{\left(x,y\right)}^{2}+G_{y}{\left(x,y\right)}^{2}},$$

where $G_{x}\left(\cdot ,\cdot \right)$ and $G_{y}\left(\cdot ,\cdot \right)$ respectively perform Sobel filtering on the horizontal and vertical gradients of the grayscale image $I_{\mathrm{gray}}$ of the original image $I_{\mathrm{RGB}}$. The $\delta_{H}$ is expressed as

(6) $$\delta_{H}=\left\{\begin{array}{cc} 1 & \frac{\sum_{x=1}^{H}\sum_{y=1}^{W}\left|F_{\mathrm{HF}}\left(x,y\right)\right|}{\sum_{x=1}^{H}\sum_{y=1}^{W}\left|F\left(x,y\right)\right|}<H_{t}\\ 0 & \mathrm{otherwise} \end{array}\right.,$$

where $H_{t}$ is the corresponding threshold, set to 0.2. $F_{\mathrm{HF}}\left(\cdot \right)$ is expressed as

(7) $$F_{\mathrm{HF}}\left(x,y\right)=F\left(x,y\right)\cdot \mathrm{Mask}\left(x,y\right),$$

where F(x,y) and Mask(x,y) are expressed as

(8) $$F\left(x,y\right)=\mathrm{FS}(\sum_{u=0}^{H-1}\sum_{v=0}^{W-1}I_{\mathrm{gray}}\left(u,v\right)\cdot e^{-2ßi(\frac{x_{u}}{H}+\frac{y_{v}}{W})}),$$

(9) $$\mathrm{Mask}\left(x,y\right)=\left\{\begin{array}{cc} 0 & \sqrt{{\left(x-c_{u}\right)}^{2}+{\left(y-c_{v}\right)}^{2}}\leq r\\ 1 & \mathrm{otherwise} \end{array}\right.,$$

where FS(·) is a frequency shift that centers the zero-frequency (DC) component, and $\mathrm{Mask}\left(x,y\right)\in {\left\{0,1\right\}}^{H\times W}$ is a circular low-pass suppression mask centered at $\left(c_{u},c_{v}\right)=\left(\frac{H}{2},\frac{W}{2}\right)$, with radius $r=\frac{\min(H,W)}{4}$.

Considering that existing blur metrics may not fully capture the spatial variations of blur, we conducted manual visual inspections of each candidate image to refine the dataset. Based on evaluations by multiple experts specializing in visual perception, we retained only those images exhibiting noticeable non-uniform blur. This process ensures that the dataset accurately reflects the complex and spatially varying blur characteristics commonly observed in real-world underwater environments.

### 3.2. Hybrid Interaction Attention Module

**Figure 4:**
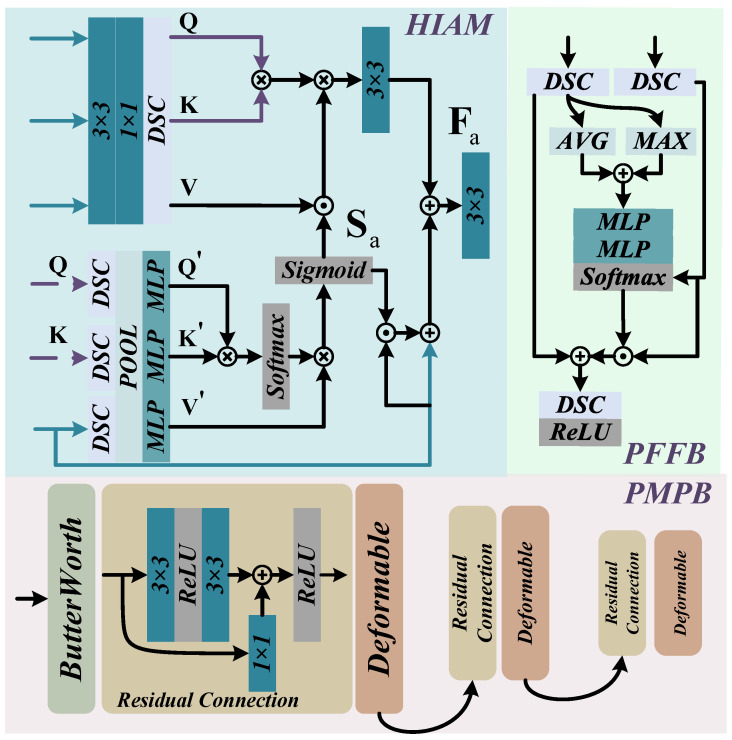
PMSPNet network components, including HIAM, PMPB, and PFFB.

To enhance the network’s capacity for deep feature extraction and precise localization of blurred regions in underwater scenes, we propose a Hybrid Interaction Attention Module (HIAM), as shown in Figure 4. Unlike traditional hybrid methods [43,44], HIAM, a cross-scale dual-channel design, utilizes an interactive cross-attention fusion dual-attention strategy for adaptive weighting control. This hybrid design enables collaborative modeling of localized blur features and global semantic structures, achieving coarse estimation of blurred feature extraction and localization.

Specifically, given an input feature $I\in R^{C\times H\times W}$, the process is represented as

(10) $$F_{a}={\mathrm{Conv}}_{3\times 3}\left(L_{a}\left(I,S_{a}\right)+G_{a}\left(Q^{\prime },K^{\prime },I\right)\right),$$

where ${\mathrm{Conv}}_{3\times 3}\left(\cdot \right)$ indicates 3×3 convolution operation. The calculation process for other characters is as follows.

Firstly, we obtain Q, K, and V as follows:

(11) $$Q,K,V=\mathrm{DSC}({\mathrm{Conv}}_{1\times 1}\left({\mathrm{Conv}}_{3\times 3}\left(I,I,I\right)\right)),$$

where ${\mathrm{Conv}}_{1\times 1}\left(\cdot \right)$ indicates 1×1 convolution operation, and DSC(·) indicates depthwise separable convolution. Subsequently, $Q^{\prime }$, $K^{\prime }$, and $V^{\prime }$ are obtained by

(12) $$Q^{\prime }=\left(P_{m}\left({\mathrm{DSC}}_{q}\left(Q\right)\right)+P_{a}\left({\mathrm{DSC}}_{q}\left(Q\right)\right)\right)W_{Q},$$

(13) $$K^{\prime }=\left(P_{m}\left({\mathrm{DSC}}_{k}\left(K\right)\right)+P_{a}\left({\mathrm{DSC}}_{k}\left(K\right)\right)\right)W_{K},$$

(14) $$V^{\prime }=\left(P_{m}\left({\mathrm{DSC}}_{v}\left(I\right)\right)+P_{a}\left({\mathrm{DSC}}_{v}\left(I\right)\right)\right)W_{V},$$

where $P_{m}\left(\cdot \right)$ and $P_{a}\left(\cdot \right)$ are adaptive global max pooling and average pooling, and $W_{Q},\;W_{K},\;W_{V}\in R^{C\times d}$ are trainable matrices. $S_{a}$ can be given by the following:

(15) $$S_{a}=\sigma_{\mathrm{sig}}\left(\sigma_{\mathrm{soft}}\left(\frac{Q^{\prime }{\left(K^{\prime }\right)}^{T}}{\sqrt{d^{\prime }}}\right)V^{\prime }\right),$$

where $\sigma_{\mathrm{sig}}\left(\cdot \right)$ and $\sigma_{\mathrm{soft}}\left(\cdot \right)$ are Sigmoid and Softmax activation functions, and $d^{\prime }$ is the dimension of the key vector. Thus, $L_{a}\left(I,S_{a}\right)$ can be expressed as

(16) $$L_{a}\left(I,S_{a}\right)={\mathrm{Conv}}_{3\times 3}\left(\frac{Q{\left(K\right)}^{T}}{\sqrt{d}}\left(V⊙S_{a}\right)\right),$$

where ⊙ is a pointwise multiplication operation. $G_{a}\left(Q^{\prime },K^{\prime },I\right)$ can be expressed as

(17) $$G_{a}\left(Q^{\prime },K^{\prime },I\right)=S_{a}⊙I+I.$$

### 3.3. Progressive Motion-Aware Perception Branch

To further enhance the modeling of aware features in underwater non-uniform blur scenarios, we introduce a Progressive Motion-Aware Perception Branch (PMPB), as depicted in the lower part of Figure 4. This module progressively refines the localization of non-uniform blur features in a coarse-to-fine manner, thereby strengthening the network’s multi-level perception of structural details.

Specifically, a two-dimensional Butterworth filter is applied to the input image to perform frequency adjustment. This operation amplifies the response of blurred regions across varying frequency components, improving the network’s sensitivity to related features [45,46]. The filtering process is defined as

(18) $$I_{b}=L2R\left(\mathrm{Concat}\left(H_{\mathrm{hf}}\left(L\right),A,B\right)\right),$$

where L2R(·) converts the image from LAB to RGB color space; Concat(·) is a channel splicing operation; and $L=I_{\mathrm{LAB}}^{\left(l\right)}$, $A=I_{\mathrm{LAB}}^{\left(a\right)}$, and $B=I_{\mathrm{LAB}}^{\left(b\right)}$ are the luminance and color channels in the LAB space, respectively. $H_{\mathrm{hf}}\left(\cdot \right)$ represents high-frequency extraction operations, expressed as

(19) $$H_{\mathrm{hf}}\left(L\right)=ℜ\left({\tilde{L}}_{\mathrm{spatial}}\right),$$

where ℜ(·) denotes the real part of a complex number. ${\tilde{L}}_{\mathrm{spatial}}$ can be expressed as

(20) $${\tilde{L}}_{\mathrm{spatial}}=F^{-1}\left({\tilde{L}}_{s}\right),$$

where $F^{-1}\cdot$ is the inverse fast Fourier transform (IFFT). ${\tilde{L}}_{s}$ can be expressed as

(21) $${\tilde{L}}_{s}=\mathrm{IS}\left(\tilde{L}\right),$$

(22) $$\tilde{L}=\hat{L}\cdot \left(1-H_{\mathrm{bw}}\right),$$

where IS(·) indicates that the centralized spectrum diagram will be restored to its original layout. $\hat{L}\in C^{B\times 1\times H\times W}$ can be expressed as

(23) $$\hat{L}=\mathrm{FS}\left(F\left(L\right)\right),$$

where F(·) is the fast Fourier transform (FFT).

The Butterworth filter $H_{\mathrm{bw}}$ is constructed as

(24) $$H_{\mathrm{bw}}\left(u,v\right)=\frac{1}{1+{\left(\frac{D_{0}}{\sqrt{{\left(u-\frac{H}{w}\right)}^{2}+{\left(v-\frac{W}{2}\right)}^{2}+\epsilon }}\right)}^{2n}},$$

where $D_{0}$ is the cutoff frequency, *n* is the filter order, and ϵ is the anti-decimation constant, set to $10^{-6}$.

Subsequently, the frequency domain enhanced image $I_{b}$ is subjected to initial feature extraction, which is denoted as

(25) $$I_{i}=\sigma_{\mathrm{re}}\left({\mathrm{Conv}}_{1\times 1}\left(I_{b}\right)+{\mathrm{Conv}}_{3\times 3}\left(\sigma_{\mathrm{re}}\left({\mathrm{Conv}}_{3\times 3}\left(I_{b}\right)\right)\right)\right),$$

where $\sigma_{\mathrm{re}}\left(\cdot \right)$ is the ReLU activation function.

Finally, we introduce Deformable Convolution [47] to adaptively adjust the sampling position for more accurate perception and feature extraction of the non-uniform blur region, which is expressed as

(26) $$I_{o}^{p_{0}}=\sum_{k=1}^{K}w_{k}\cdot \left(\sum_{q\in N(p_{0}+p_{k}+△p_{k})}G\left(q,p_{0}+p_{k}+△p_{k}\right)\cdot I_{i}^{q}\right),$$

where $I_{o}^{p_{0}}$ is the value of the output feature map at position $p_{0}$; $w_{k}$ is the weight of the kth position of the convolution kernel; N is a bilinear interpolating neighborhood; G(q,·) is the interpolated weight, satisfying $\sum_{q}G\left(q,\cdot \right)=1$; $p_{k}$ is the kth standard sampling offset in the convolution kernel; $△p_{k}$ is the learnable offset of the kth position; and $I_{i}^{q}$ is the value of the input feature $I_{i}$ at position *q*.

The fine features are fed into the backbone to provide it with feature guidance, which is expressed as

(27) $$F_{a}=I_{o}+{\mathrm{Conv}}_{3\times 3}\left(\mathrm{Concat}\left(F_{a},I_{o}\right)\right).$$

### 3.4. Progressive Feature Feedback Block

To achieve lossless extraction of hierarchical semantic features during reconstruction, we propose a Progressive Feature Feedback Block (PFFB), as illustrated in Figure 4. This module facilitates inter-layer information interaction by constructing a hierarchical feedback pathway, allowing recovered features from subsequent stages to guide and refine the representation learning in previous stages. The structure of the PFFB is represented as

(28) $$F_{f}=\sigma_{\mathrm{re}}\left({\mathrm{DSC}}_{1}\left(F_{\mathrm{att}}+F_{m}\right)\right),$$

where $F_{\mathrm{att}}$ can be expressed as

(29) $$F_{\mathrm{att}}=\sigma_{\mathrm{soft}}\left({\mathrm{MLP}}_{1,2}\left(P_{m}\left(F_{m}\right)+P_{m}\left(F_{m}\right)\right)\right)⊙{\mathrm{DSC}}_{2}\left(F_{\mathrm{up}}+F_{\mathrm{cu}}\right),$$

where $F_{m}={\mathrm{DSC}}_{3}\left(F_{\mathrm{up}}+F_{\mathrm{cu}}\right)$, ${\mathrm{MLP}}_{1,2}\left(\cdot \right)$ is a multilayer perceptron, and $F_{\mathrm{up}}$ and $F_{\mathrm{cu}}$ are the previous and current level recovery features in the decoder, respectively.

### 3.5. Loss Function

To increase visual quality and perceptual fidelity of the restored image, we design a composite loss function that jointly enforces constraints on color accuracy, structural consistency, frequency response, and perceptual realism. This multi-objective formulation ensures that the restored images align closely with ground truth data and exhibit enhanced perceptual quality. The overall loss is defined as

(30) $$L_{t}=L_{\mathrm{charb}}+\lambda_{\mathrm{fft}}\cdot L_{\mathrm{fft}}+\lambda_{\mathrm{lab}}\cdot L_{\mathrm{lab}}+\lambda_{\mathrm{lch}}\cdot L_{\mathrm{lch}}+L_{\mathrm{vgg}}+L_{\mathrm{color}},$$

where $L_{\mathrm{charb}}$, $L_{\mathrm{fft}}$, $L_{\mathrm{lab}}$, $L_{\mathrm{lch}}$, $L_{\mathrm{vgg}}$, and $L_{\mathrm{color}}$ are charbonnier loss, fast fourier transform loss, LAB color loss, LCH color loss, perceptual loss, and color constancy loss, respectively. $\lambda_{\mathrm{fft}}$, $\lambda_{\mathrm{lab}}$, and $\lambda_{\mathrm{lch}}$ are the corresponding weighting coefficients, set to $1\times 10^{-1}$, $1\times 10^{-6}$, and $1\times 10^{-2}$, respectively. $L_{\mathrm{charb}}$ is defined as

(31) $$L_{\mathrm{charb}}\left(x,y\right)=\frac{1}{N}\sum_{i=1}^{N}\sqrt{{\left(x_{i}-y_{i}\right)}^{2}+\epsilon^{2}},$$

where *x* and *y* are the predicted image and ground truth, and *N* is the total number of pixels in the image. $L_{\mathrm{fft}}$ is defined as

(32) $$L_{\mathrm{fft}}\left(x,y\right)={∥F(x)-F(y)∥}_{1},$$

where $\left|\right|\cdot {\left|\right|}_{1}$ is L1 loss. $L_{\mathrm{lab}}$ is defined as

(33) $$L_{\mathrm{lab}}\left(x,y\right)=L_{l}\left(x,y\right)+\alpha \cdot L_{\mathrm{ab}}\left(x,y\right),$$

where α is the weighting coefficient, set to 1. $L_{l}\left(\cdot \right)$ and $L_{\mathrm{ab}}\left(\cdot \right)$ are the brightness and color channel loss in the LAB channel, defined as

(34) $$L_{l}\left(x,y\right)=\frac{1}{N}\sum_{i=1}^{N}\left|L_{x}^{\left(i\right)}-L_{y}^{\left(i\right)}\right|,$$

(35) $$L_{\mathrm{ab}}\left(x,y\right)=\frac{1}{N}\sum_{i=1}^{N}\sum_{k=1}^{N}P_{y}^{\left(i,k\right)}\cdot \log\left(\frac{P_{y}^{\left(i,k\right)}}{P_{x}^{\left(i,k\right)}+\epsilon }\right)\approx -\frac{1}{N}\sum_{i=1}^{N}\sum_{k=1}^{N}P_{y}^{\left(i,k\right)}\cdot \log\left(P_{x}^{\left(i,k\right)}+\epsilon \right),$$

where $P_{x}^{\left(i,k\right)}$ and $P_{y}^{\left(i,k\right)}$ are the softmax weights for the *k*th bin at the *i*th pixel position of images *x* and *y*. $L_{\mathrm{lch}}$ is defined as

(36) $$L_{\mathrm{lch}}=L_{\mathrm{ll}}+L_{\mathrm{lc}}+L_{\mathrm{lh}},$$

where $L_{\mathrm{ll}}$ and $L_{\mathrm{lc}}$ represent brightness and chromaticity L1 loss (reference Equation (34)), and $L_{\mathrm{lh}}$ represents hue distribution loss (reference Equation (35)) in LCH color space.

In addition, $L_{\mathrm{vgg}}$ utilizes the multi-scale features of the VGG19 network to measure the differences between the enhanced image and the reference image in the high-level semantic space. $L_{\mathrm{color}}$ achieves color balance consistency by minimizing the pairwise Euclidean distances between the *R*, *G*, and *B* channels.

## 4. Experiments

### 4.1. Datasets

We train and evaluate the proposed network using the constructed N2UD dataset and compare its performance against state-of-the-art methods on N2UD and three publicly available underwater image enhancement benchmarks: EUVP [48], LSUI [49], and UIEB [50]. Additionally, to assess the practical benefits of the enhanced images in real-world scenarios, we conduct downstream task evaluations on the DUO dataset [51], demonstrating the model’s effectiveness in supporting higher-level underwater vision tasks.

#### 4.1.1. N2UD

The N2UD dataset comprises 3201 real underwater image pairs, each consisting of a non-uniformly blurred image and its corresponding reference. Among these, 2246 pairs were used for training, 624 for testing, and 322 for validation. The dataset consists of several publicly available underwater image enhancement datasets. Using a combination of quantitative blur metrics with manual visual inspection, we screened high-quality non-uniform blur image pairs. Specifically, the dataset includes 555 images from the EUVP dataset, 2190 from the LSUI dataset, and 465 from the UIEB dataset. The selected images span a range of resolutions, from 256×256 to 1280×720, and diverse underwater environments and imaging conditions. The dataset is available at https://github.com/UI2025/N2UD, accessed on 28 August 2025.

#### 4.1.2. EUVP

The EUVP dataset is designed to support the enhancement of color and structural details in real-world underwater images. It employs multiple underwater sensors (GoPros, Aqua AUV’s uEye cameras, low-light USB cameras, and Trident ROV’s HD camera) to capture underwater images and conduct marine exploration and human-robot cooperative experiments in different locations under various visibility conditions. It contains both paired and unpaired collections of approximately 20,000 images, covering a broad spectrum of underwater environments, lighting conditions, and viewpoints. The dataset includes both real and synthetically degraded underwater images at various resolutions, making it suitable for training and evaluating deep learning-based underwater image enhancement models. The dataset is available at https://irvlab.cs.umn.edu/resources/euvp-dataset, accessed on 17 May 2025.

#### 4.1.3. LSUI

The LSUI dataset, filtered and screened based on various real underwater image data, is a large-scale collection of 4279 real-world underwater images captured across multiple scenes, including shallow waters, deep sea, coral reefs, and shipwrecks. It encompasses a wide variety of conditions in terms of lighting, water quality, and color degradation. LSUI emphasizes real data distributions and high scene complexity, making it well-suited for studying naturally occurring underwater degradations. The dataset is available at https://lintaopeng.github.io/_pages/UIE%20Project%20Page.html, accessed on 17 May 2025.

#### 4.1.4. UIEB

The UIEB dataset collects and processes underwater images on different platforms and includes private underwater shooting videos. It is one of the most widely used benchmarks in underwater image enhancement. It includes 890 real-world underwater images of varying resolutions, most of which have been enhanced by domain experts to form a high-quality paired dataset. It is commonly used for both subjective and objective evaluation of enhancement algorithms. In addition, UIEB includes a challenging subset, Challenging-60, comprising 60 challenging unpaired samples used to test algorithmic robustness under extreme conditions. The dataset is available at https://li-chongyi.github.io/proj_benchmark.html, accessed on 17 May 2025.

#### 4.1.5. DUO

The DUO dataset is a high-quality benchmark designed for underwater object detection (UOD) tasks. These data were obtained by collecting underwater images through underwater photography and labeling them, and they were used to study the perception decisions and sensor sensitivity issues of underwater robots. It contains 7782 images captured from diverse underwater environments, including shallow and deep water, turbid regions, and varied lighting conditions. The dataset features a wide array of target types, including starfish, sea urchins, and fish, along with substantial variation in scene structure, imaging quality, and target scale. All images are provided at a uniform resolution of 1920×1080, with 6671 images allocated for training and 1111 for testing. In this work, we utilize the trained enhancement model to augment the DUO dataset and perform downstream detection tasks, thereby validating the positive influence of our method on higher-level vision applications. The dataset is available at https://github.com/chongweiliu/DUO, accessed on 18 May 2025.

### 4.2. Experimental Configuration

#### 4.2.1. Implementation Details

The proposed network was implemented using the PyTorch 2.0.1 framework and trained on a Linux-based system equipped with a GeForce RTX 3090 GPU (24 GB), 250 GB of memory, and 80 Intel(R) Xeon(R) Gold 5218R processors (2.10 GHz). The training was performed using the Adam optimizer with an initial learning rate of $1\times 10^{-4}$. A cosine learning rate scheduling strategy with a warm-up phase was employed, in which the learning rate was gradually increased during the first 10 epochs, after which it followed a cosine decay schedule. The network was trained for 200 epochs with a batch size of 8. To improve the model’s robustness and generalization capability, standard data augmentation techniques such as random horizontal and vertical flipping were applied during training. To prevent overfitting and ensure stable convergence, an early stopping mechanism was employed, in which training was terminated if the validation loss failed to improve for 10 consecutive epochs. Furthermore, random seeds were fixed across all experiments to guarantee reproducibility of the reported results.

#### 4.2.2. Evaluation Metrics

To comprehensively assess the performance of the proposed method in restoring underwater images affected by non-uniform blur, we employed a diverse set of evaluation metrics covering full-reference, no-reference, perceptual, and sharpness-based aspects. For full-reference evaluation, we used the Peak Signal-to-Noise Ratio (PSNR), the Structural Similarity Index (SSIM), and the Feature Similarity Index (FSIM) to quantify pixel-level fidelity and structural consistency between the restored images and their ground-truth counterparts. To evaluate perceptual quality, we adopted LPIPS [52], which measures perceptual similarity using deep feature representations and aligns better with human visual perception.

For no-reference evaluation, we employed underwater-specific quality metrics, including UIQM [53] and UCIQE [54], to evaluate key visual attributes such as color fidelity, contrast, and clarity. Additionally, we introduced NIQE [55], a natural image quality evaluator, and URanker [56], a human preference-based perceptual scoring model, to further assess image naturalness and subjective quality. To evaluate the preservation of fine details and edge sharpness, we incorporated high-frequency sharpness metrics, including Laplacian variance, Tenengrad, and Brenner gradient, each capturing edge clarity and detail recovery from different computational perspectives.

The integration of these complementary metrics enables a comprehensive and objective evaluation of image enhancement performance, ensuring the model’s robustness and practical applicability in real-world underwater scenarios and downstream tasks.

### 4.3. Performance Comparison

#### 4.3.1. N2UD

To comprehensively evaluate the effectiveness of the proposed method, we conducted extensive experiments on the constructed N2UD dataset using both full-reference and no-reference image quality assessment metrics. The results are presented in Table 1 and Table 2.

As shown in Table 1, traditional image processing methods exhibit generally poor performance across full-reference metrics. Specifically, their PSNR values typically fall below 18 dB, and LPIPS scores often exceed 0.3, indicating significant limitations in perceptual quality and visual fidelity. These methods struggle to adaptively model complex blur degradation, which severely limits their scalability in real-world applications. Furthermore, traditional methods are often computationally intensive and inefficient. Some require up to 1.24 s to process a single image, rendering them unsuitable for time-sensitive tasks. In contrast, deep learning-based UIE methods demonstrate more robust performance. GAN-based approaches offer modest improvements in some metrics. CycleGAN achieves relatively high SSIM (0.83±0.11) and LPIPS (0.24±0.01) compared to traditional methods. However, it exhibits considerable PSNR variance (±4.72), highlighting a lack of consistency and reliability in generative adversarial models. U-Shape, leveraging a multi-scale architecture, performs well across all full-reference metrics but still falls short of the performance achieved by PMSPNet. Among recently proposed Transformer-based methods, HistFormer suffers from mode collapse issues, leading to degraded performance across multiple metrics. Additionally, it incurs a high computational cost (44.42 GFLOPs) and slower inference speeds. PhaseFormer, in contrast, shows a more balanced performance. However, its relatively low SSIM indicates limitations in preserving structural integrity. PUIE-Net achieves favorable results across most metrics, yet its complexity is a concern, with computational overhead reaching 150.69 GFLOPs and inference time extending to 0.13 s per image. SGUIE performs well in FSIM and LPIPS, reflecting strengths in perceptual quality. Nonetheless, its overall effectiveness remains inferior when compared to PMSPNet. PMSPNet achieves superior performance in both objective metrics and computational efficiency, confirming its robustness and practical applicability for real-world underwater deblurring tasks.

As shown in the no-reference image quality evaluation results in Table 2, although some methods perform reasonably well on individual metrics, a noticeable gap remains in overall quality. Notably, PMSPNet consistently leads across multiple perceptual quality metrics, such as UIQM and UCIQE, indicating that its enhanced images exhibit more natural color, clarity, and contrast, aligning well with human visual preferences. Interestingly, some traditional methods achieve higher scores on sharpness-based metrics, such as Tenengrad and Brenner. This contrasts with their overall inferior performance on full-reference and other no-reference metrics. The root cause of this “contradiction” is that many current reference-free metrics capture only specific local attributes, failing to reflect comprehensive image quality or perceptual realism.

**Figure 5:**
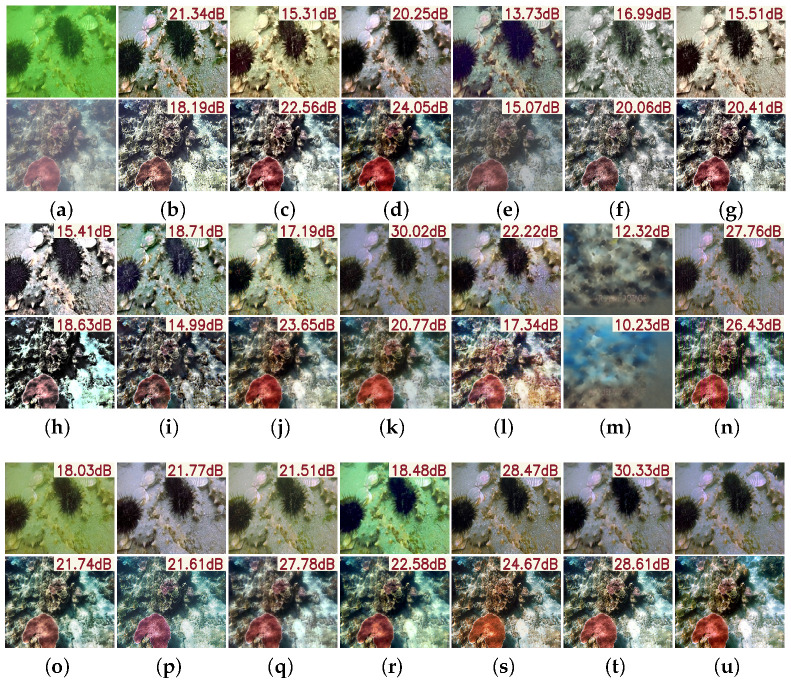
Visual comparison on the N2UD dataset, with the PSNR value of the image shown in the upper-right corner of the image. (**a**) Raw images. (**b**) WFAC [25]. (**c**) WWPF [57]. (**d**) HFM [58]. (**e**) HLRP [59]. (**f**) ACDC [60]. (**g**) MMLE [61]. (**h**) PCDE [62]. (**i**) TEBCF [63]. (**j**) CycleGAN [29]. (**k**) U-Shape [49]. (**l**) FUnIE-GAN [48]. (**m**) Histoformer [64]. (**n**) Phaseformer [65]. (**o**) UIR-PolyKernel [66]. (**p**) CCL-Net [67]. (**q**) PUIE-Net [68]. (**r**) USUIR [69]. (**s**) SGUIE [70]. (**t**) PMSPNet. (**u**) Ground Truth.

The vision comparisons in Figure 5 further support this conclusion. While traditional methods can enhance local edge sharpness, they often suffer from over-sharpening, resulting in structural distortions, unnatural textures, or even pattern collapse, leading to a visually unrealistic “pseudo-enhancement” effect. In contrast, deep learning-based methods leverage strong feature representation and adaptive learning to restore degraded details more comprehensively. By capturing complex, non-uniform blur patterns across multiple scales and dimensions, PMSPNet achieves accurate regional restoration while preserving structural clarity and avoiding artifacts. This results in improved naturalness, semantic consistency, and stronger generalization in real-world conditions.

**Figure 6:**
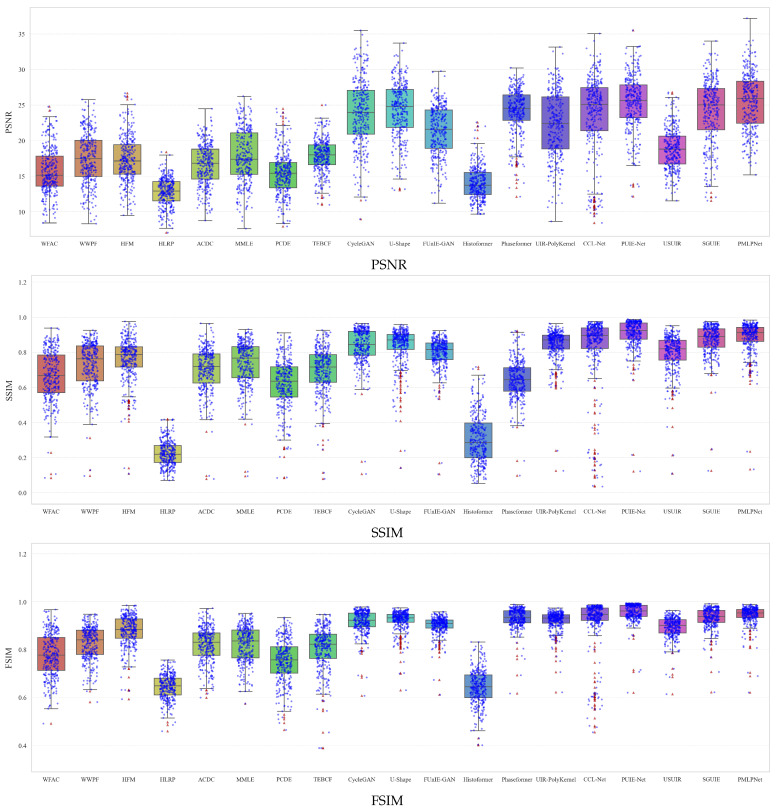
Box plot comparison of PSNR, SSIM, and FSIM on the N2UD dataset.

To further validate model robustness, Figure 6 presents the statistical distribution of three representative full-reference metrics. Traditional methods exhibit low and tightly clustered scores, indicating limited and inconsistent enhancement. Deep learning-based methods perform better overall. PMSPNet shows the highest median and maximum values, especially exceeding 35 dB in PSNR, and maintains stable, high distributions in SSIM and FSIM. The low dispersion of PMSPNet scores reflects its adaptability across various scenes, attributed to its hybrid interaction attention and progressive motion-aware modules, which jointly enhance spatially non-uniform blur modeling and semantic detail recovery.

#### 4.3.2. EUVP

To further assess the generalization capability of the proposed network, we evaluated pre-trained models on the EUVP dataset. As shown in Table 3, PMSPNet achieves the best performance across nearly all full-reference metrics, including PSNR (25.81), SSIM (0.85), and FSIM (0.94), and the lowest LPIPS (0.21), demonstrating superior fidelity and perceptual quality. In no-reference perceptual assessments, PMSPNet remains competitive, achieving a UIQM of 3.09, a UCIQE of 0.36, and a URanker of 1.16, consistently outperforming some existing methods.

**Figure 7:**
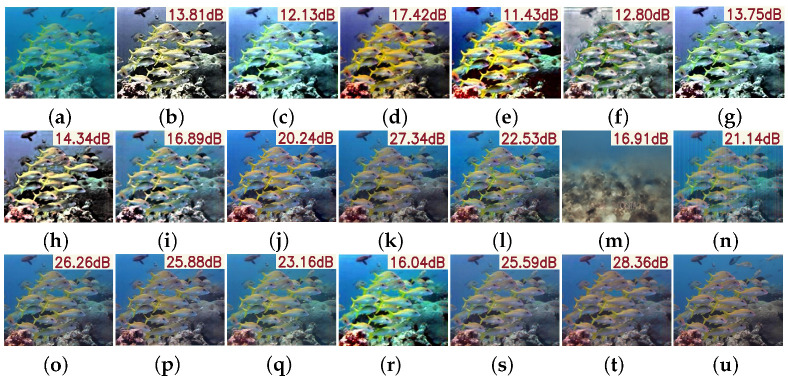
Visual comparison on the EUVP dataset, with the PSNR value of the image shown in the upper-right corner of the image. (**a**) Raw image. (**b**) WFAC [25]. (**c**) WWPF [57]. (**d**) HFM [58]. (**e**) HLRP [59]. (**f**) ACDC [60]. (**g**) MMLE [61]. (**h**) PCDE [62]. (**i**) TEBCF [63]. (**j**) CycleGAN [29]. (**k**) U-Shape [49]. (**l**) FUnIE-GAN [48]. (**m**) Histoformer [64]. (**n**) Phaseformer [65]. (**o**) UIR-PolyKernel [66]. (**p**) CCL-Net [67]. (**q**) PUIE-Net [68]. (**r**) USUIR [69]. (**s**) SGUIE [70]. (**t**) PMSPNet. (**u**) Ground Truth.

As illustrated in Figure 7, visual comparisons further substantiate PMSPNet’s advantages. Compared to prior methods, PMSPNet produces images with sharper edges, more natural color reproduction, and enhanced contrast. Traditional methods such as HLRP, ACDC, and MMLE suffer from color shifts, amplified noise, or loss of structural detail. Although deep learning methods like CycleGAN and U-Shape show improved enhancement, they still exhibit color deviations or insufficient deblurring in complex scenes. In contrast, PMSPNet effectively restores clarity and color fidelity even under severe degradation, delivering results that are perceptually closest to the ground truth.

#### 4.3.3. LSUI

Table 4 presents the evaluation results on the LSUI dataset. PMSPNet achieves the highest scores across key full-reference metrics, along with the lowest LPIPS, indicating superior fidelity and perceptual quality in the restored images. In the no-reference evaluation, PMSPNet also ranks first in UIQM and second in UCIQE, further demonstrating its effectiveness in enhancing visual quality. In contrast, traditional methods and other deep learning-based approaches show reduced performance across most metrics due to their limited capacity to manage complex color degradation and structural blurring prevalent in underwater environments.

**Figure 8:**
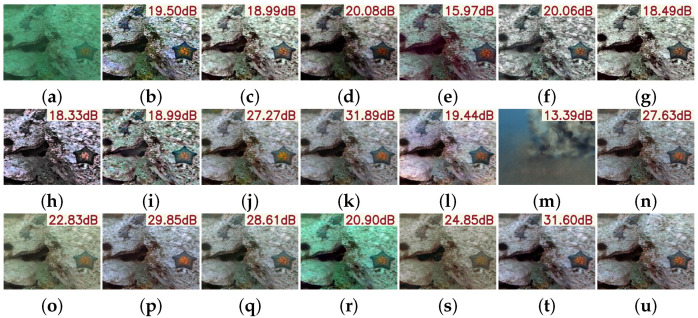
Visual comparison on the LSUI dataset, with the PSNR value of the image shown in the upper-right corner of the image. (**a**) Raw image. (**b**) WFAC [25]. (**c**) WWPF [57]. (**d**) HFM [58]. (**e**) HLRP [59]. (**f**) ACDC [60]. (**g**) MMLE [61]. (**h**) PCDE [62]. (**i**) TEBCF [63]. (**j**) CycleGAN [29]. (**k**) U-Shape [49]. (**l**) FUnIE-GAN [48]. (**m**) Histoformer [64]. (**n**) Phaseformer [65]. (**o**) UIR-PolyKernel [66]. (**p**) CCL-Net [67]. (**q**) PUIE-Net [68]. (**r**) USUIR [69]. (**s**) SGUIE [70]. (**t**) PMSPNet. (**u**) Ground Truth.

The visual results in Figure 8 further highlight the advantages of PMSPNet. Traditional algorithms often leave residual blur or introduce significant color distortions. While learning-based models such as U-Shape and CCL-Net show improved results, they still suffer from over-smoothing and color shifts, particularly in fine details. In comparison, PMSPNet effectively recovers key textures, preserves natural color tones, and enhances contrast, producing visually more realistic and aesthetically pleasing results.

#### 4.3.4. UIEB

The UIEB dataset presents a broad spectrum of underwater imaging challenges, including severe color distortion, low contrast, and complex lighting conditions. As shown in Table 5, PMSPNet outperforms most compared methods across full-reference and no-reference metrics. Specifically, it achieves a PSNR of 22.43 dB, SSIM of 0.86, and a low LPIPS of 0.21, reflecting strong reconstruction accuracy and perceptual similarity. While methods like CCL-Net and SGUIE yield competitive results, their higher variance in metrics such as URanker and UIQM suggests inconsistent performance. In contrast, PMSPNet delivers high-quality results, demonstrating robustness across varying underwater degradation types.

**Figure 9:**
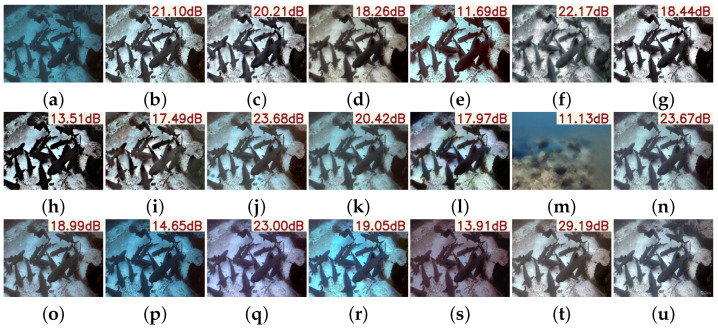
Visual comparison on the UIEB dataset, with the PSNR value of the image shown in the upper-right corner of the image. (**a**) Raw image. (**b**) WFAC [25]. (**c**) WWPF [57]. (**d**) HFM [58]. (**e**) HLRP [59]. (**f**) ACDC [60]. (**g**) MMLE [61]. (**h**) PCDE [62]. (**i**) TEBCF [63]. (**j**) CycleGAN [29]. (**k**) U-Shape [49]. (**l**) FUnIE-GAN [48]. (**m**) Histoformer [64]. (**n**) Phaseformer [65]. (**o**) UIR-PolyKernel [66]. (**p**) CCL-Net [67]. (**q**) PUIE-Net [68]. (**r**) USUIR [69]. (**s**) SGUIE [70]. (**t**) PMSPNet. (**u**) Ground Truth.

As illustrated in Figure 9, PMSPNet excels at restoring fine structures and maintaining natural color tones, even in severely degraded scenarios. Compared to methods like UIR-PolyKernel and HistFormer, which often produce over-smoothed textures or color shifts, PMSPNet effectively reconstructs edges and preserves the texture of marine organisms. It also avoids common artifacts, such as excessive blue saturation or overly enhanced contrast, frequently observed in other approaches. This balance across metrics highlights the visual fidelity and realism of PMSPNet, making it both quantitatively superior and perceptually compelling.

In summary, the above experiments demonstrate that PMSPNet can effectively remove underwater non-uniform blur, generating clearer structures, more natural colors, and fewer artifacts and exhibiting strong generalization across diverse underwater scenes. Compared to both traditional and deep learning-based baselines, PMSPNet achieves an optimal trade-off between detail preservation and deblurring, especially under challenging conditions, validating the effectiveness and robustness of the proposed approach.

### 4.4. Ablation Study

To validate the effectiveness of the proposed components in PMSPNet, we conduct comprehensive ablation experiments by removing key modules and evaluating the impact of different loss function combinations. Furthermore, we assess the scalability of the proposed algorithm in downstream tasks.

#### 4.4.1. Butterworth Filter

**Figure 10:**
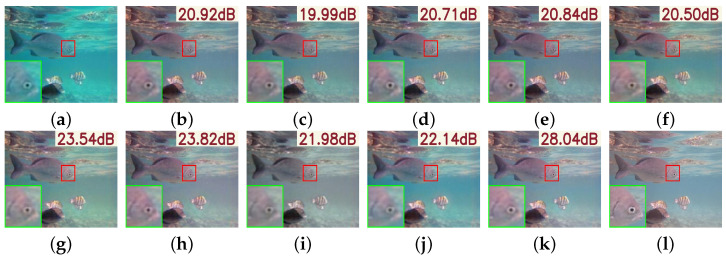
Visual comparison of networks using different components and loss functions. PSNR is shown in the top-right, and the bottom-left presents a zoomed-in view of the red-boxed region. (**a**) Raw image. (**b**) w/o Butterworth. (**c**) w/o Deformable Convolution. (**d**) w/o PFFB. (**e**) w/o PMPB. (**f**) w/o FFT loss. (**g**) w/o FFT and LAB loss. (**h**) w/o FFT, LAB and LCH loss. (**i**) w/o FFT, LAB, LCH, and VGG loss. (**j**) w/o FFT, LAB, LCH, VGG, and Color loss. (**k**) PMSPNet. (**l**) Ground Truth.

We introduce a Butterworth filter in the input stage of the PMPB to improve the frequency correspondence and mitigate the aliasing phenomenon. As shown in Table 6, although removing the module results in a slight increase in PSNR by 0.47, all other evaluation metrics exhibit a decline. In particular, SSIM drops to 0.88, UIQM decreases to 3.08, and UCIQE falls to 0.39. These results underscore the module’s effectiveness in enhancing structural clarity and perceptual quality as perceived by the human visual system. As illustrated in Figure 10b, the absence of this module leads to noticeable blurring and color artifacts, particularly around object edges and in background regions. This further confirms the module’s critical role in improving the overall structural integrity of the image and enhancing the network’s sensitivity to spatially non-uniform blur.

#### 4.4.2. Deformable Convolution

The high dynamics of deformable convolution enable precise localization and directional modeling of non-uniform blur, allowing the network to effectively capture regionally continuous blur patterns and apply accurate weighting to key features. As shown in Table 6, the removal of deformable convolution results in a substantial decline across all evaluation metrics, with PSNR dropping sharply to 17.18, indicating a significant reduction in reconstruction quality. Furthermore, the network’s overall stability is noticeably compromised. As illustrated in Figure 10c, the network without this module struggles to preserve object boundaries and spatial consistency, and the residual blur remains largely unaddressed. These results demonstrate that deformable convolution significantly enhances the network’s capacity to identify critical image features and improves the modeling of local structures, which is essential for effective UIE under non-uniform degradation conditions.

#### 4.4.3. Progressive Motion-Aware Perception Branch

The PMPB integrates a Butterworth filter and deformable convolution to progressively capture the spatial distribution of blur and guide feature extraction in the backbone, enabling a coarse-to-fine refinement of blur localization. As shown in Table 6, while the removal of PMPB results in a slight increase in PSNR, it reduces the overall stability of the network. This observation also validates the complementary roles of HIAM and PMPB: HIAM facilitates multi-directional feature perception and coarse localization of blur, whereas PMPB further refines this localization, enhancing the network’s generalization and robustness. Additionally, the observed decline in metrics such as SSIM confirms PMPB’s contribution to structural clarity and perceptual quality. As illustrated in Figure 10d, the absence of PMPB increases color distortion and reduces image sharpness, particularly in fine structures. These results underscore the importance of PMPB in preserving texture details and scale-aware representations, achieving a critical balance between global semantic understanding and local detail restoration.

#### 4.4.4. Progressive Feature Feedback Block

The PFFB hierarchically integrates multi-level features by facilitating cross-layer feedback, enabling the fusion of spatial details with deep semantic representations. As shown in Table 6, the removal of this module leads to increased information loss during image reconstruction, which diminishes the network’s adaptive reconstruction capability, resulting in a consistent, albeit modest, degradation across all evaluation metrics. Figure 10e reveals noticeable artifacts and diminished smoothness, particularly along background contours, indicating the loss of critical structural details. These results demonstrate that PFFB plays a vital role in regulating cross-layer information flow, guiding adaptive feature reweighting, and reducing reconstruction-induced information loss. Consequently, it contributes significantly to improving structural fidelity and perceptual consistency in enhanced underwater images.

#### 4.4.5. Loss Function

We investigated the impact of different loss components on the overall performance of the proposed network. As illustrated in Table 7, removing most auxiliary losses leads to a notable decline across multiple evaluation metrics. Among these, the FFT, LAB, and LCH loss functions contribute most significantly to performance improvement, enhancing objective image quality and perceptual fidelity. In particular, the inclusion of the LAB loss enhances network stability, underscoring the importance of multi-domain color perception. While the VGG and Color losses did not yield substantial improvements in numerical metrics, they contributed positively to visual quality by enhancing hue perception and overall appearance. Figure 10f–k demonstrates that models trained without specific loss terms suffer from excessive smoothing or unnatural color shifts. In contrast, the complete loss configuration consistently generates underwater images with sharper edges, more balanced color tones, and enhanced visual appeal, validating the effectiveness of each component.

#### 4.4.6. Blur Perceptual Localization

**Figure 11:**
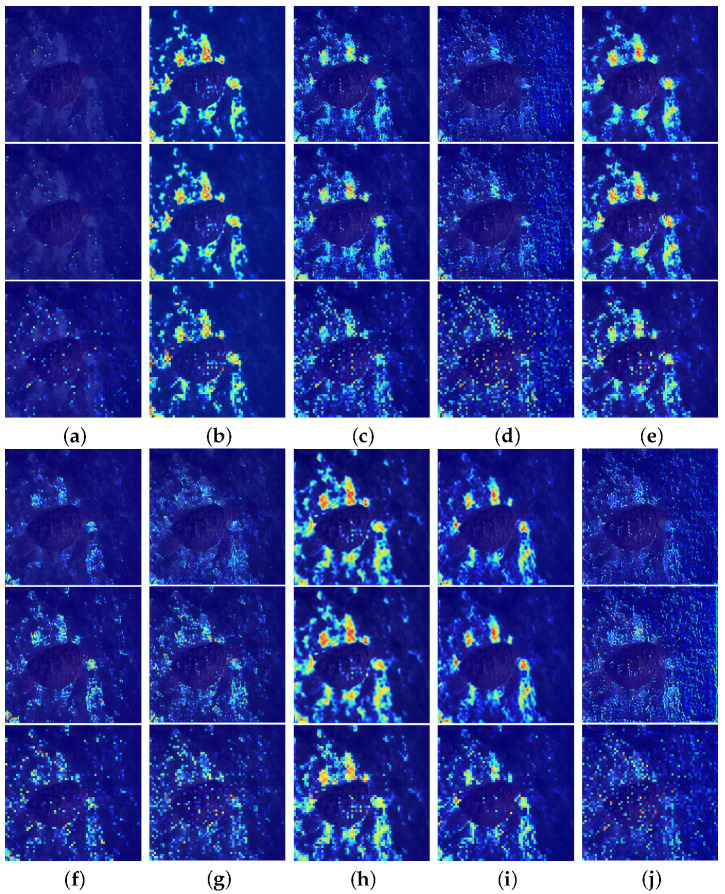
Visualization of blur localization under different components and loss functions. Each group of three images corresponds to the enhanced outputs at three different scales. (**a**) w/o Butterworth. (**b**) w/o Deformable Convolution. (**c**) w/o PFFB. (**d**) w/o PMPB. (**e**) w/o FFT loss. (**f**) w/o FFT and LAB loss. (**g**) w/o FFT, LAB, and LCH loss. (**h**) w/o FFT, LAB, LCH, and VGG loss. (**i**) w/o FFT, LAB, LCH, VGG, and Color loss. (**j**) PMSPNet.

To further investigate the contribution of components to blur perception, we visualize the heatmaps generated under various ablation settings in Figure 11, using the second column of Figure 1 as a representative example. When the Butterworth filter is removed, the heatmap exhibits higher noise levels, indicating a weakened ability to extract key features and accurately capture blur-related information. The removal of deformable convolution and the PFFB markedly impairs the network’s capacity for accurately identifying and localizing blurred regions. These components are essential for enabling coarse-to-fine blur localization and iterative attention modulation. Eliminating the PMPB module causes the network’s attention to drift while demonstrating the capability of HIAM in achieving coarse localization of blur features.

From the perspective of loss functions, removing the FFT loss impairs the network’s ability to capture frequency-domain information, thereby diminishing its capacity to identify and localize blur across multiple spatial domains. VGG, LAB, and LCH losses contribute to guiding the network’s attention toward perceptually and semantically important regions, facilitating more accurate blur localization. While Color loss has a relatively smaller effect on network performance, it plays a crucial role in suppressing over-sharpening, thereby enhancing visual naturalness.

In contrast, the proposed model exhibits the most concentrated and semantically accurate activation responses, highlighting blurred regions. These results validate the effectiveness of our architectural design and multi-component loss in improving the network’s capability for blur perception and structural fidelity restoration in underwater environments.

#### 4.4.7. Downstream Task Evaluation

To further validate the practical benefits of the proposed method, we conducted evaluations on downstream tasks, including classic feature extraction and underwater object detection (UOD).

**Figure 12:**
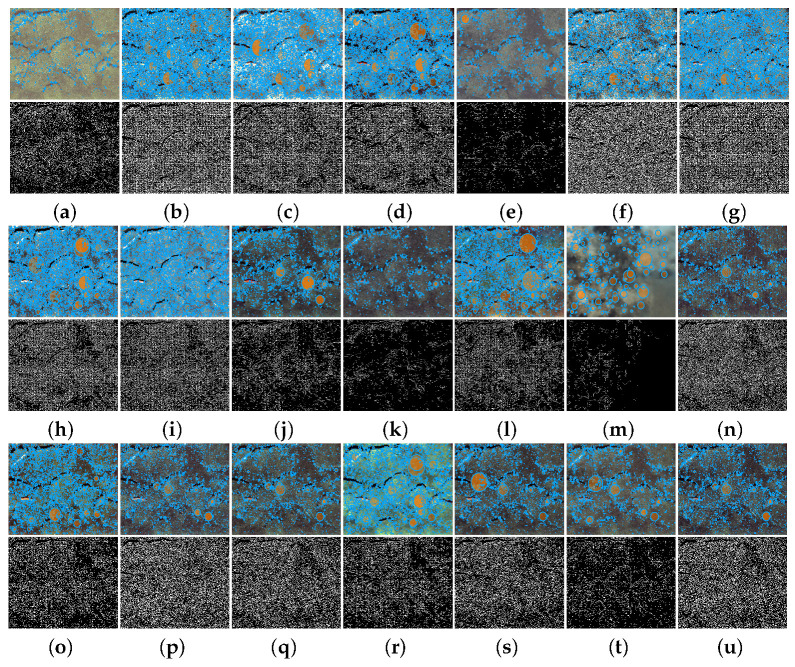
Visual comparison of SIFT keypoint and Canny edge detection results. The first row presents the visualization of SIFT keypoints, while the second row displays the corresponding Canny edge detection results. (**a**) Raw image. (**b**) WFAC [25]. (**c**) WWPF [57]. (**d**) HFM [58]. (**e**) HLRP [59]. (**f**) ACDC [60]. (**g**) MMLE [61]. (**h**) PCDE [62]. (**i**) TEBCF [63]. (**j**) CycleGAN [29]. (**k**) U-Shape [49]. (**l**) FUnIE-GAN [48]. (**m**) Histoformer [64]. (**n**) Phaseformer [65]. (**o**) UIR-PolyKernel [66]. (**p**) CCL-Net [67]. (**q**) PUIE-Net [68]. (**r**) USUIR [69]. (**s**) SGUIE [70]. (**t**) PMSPNet. (**u**) Ground Truth.

As illustrated in Figure 12, we applied SIFT keypoint detection and Canny edge detection to assess the influence of enhancement on low-level visual features. The first row shows the SIFT results, while the second row displays the Canny edges. Traditional methods often fail to preserve sufficient structural detail, introducing noise and blurring in non-salient regions, which results in some key edges being obscured or missing. In contrast, images enhanced by PMSPNet exhibit clear contours and rich edge information, closely matching the ground truth, demonstrating that PMSPNet effectively enhances geometric clarity, facilitating better feature localization.

**Figure 13:**
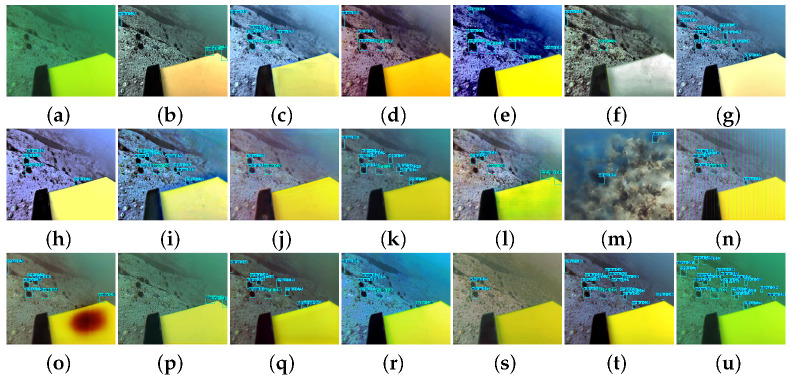
Comparison of YOLOv12 detection effects on enhanced underwater images from the DUO dataset. (**a**) Raw image. (**b**) WFAC [25]. (**c**) WWPF [57]. (**d**) HFM [58]. (**e**) HLRP [59]. (**f**) ACDC [60]. (**g**) MMLE [61]. (**h**) PCDE [62]. (**i**) TEBCF [63]. (**j**) CycleGAN [29]. (**k**) U-Shape [49]. (**l**) FUnIE-GAN [48]. (**m**) Histoformer [64]. (**n**) Phaseformer [65]. (**o**) UIR-PolyKernel [66]. (**p**) CCL-Net [67]. (**q**) PUIE-Net [68]. (**r**) USUIR [69]. (**s**) SGUIE [70]. (**t**) PMSPNet. (**u**) Detection effect of raw image.

To assess high-level perception, we applied YOLOv12 [71] for underwater object detection. A YOLOv12 model pre-trained on the original DUO dataset was used to detect objects in images enhanced by different methods. As shown in Figure 13, traditional enhancement approaches often introduce significant color distortions, which impair detection performance. Deep learning-based baselines exhibit limited performance in small object detection, primarily due to underperformance in detail preservation. In contrast, PMSPNet-enhanced images have more accurate and stable detection outcomes, achieving higher confidence scores and better alignment with the original detection results. Notably, because YOLOv12 was trained on the original images, detection performance is best in raw images.

The above results demonstrate that PMSPNet not only improves perceptual image quality but also preserves semantic fidelity, making it well-suited for integration into real-world underwater robotic systems and visual monitoring pipelines.

#### 4.4.8. Limitation Analysis

Although PMSPNet demonstrates superior performance in non-uniform underwater image deblurring, several limitations remain. Due to the incorporation of multi-scale perception and progressive feedback mechanisms, the model introduces additional computational overhead, which may restrict its real-time deployment on extremely resource-constrained underwater robotic platforms. While the proposed N2UD dataset covers diverse non-uniform blur patterns, it still cannot fully represent the wide spectrum of degradations in real-world underwater environments, such as extreme turbidity, lighting fluctuations, or dynamic background interference. While we validated the universality of PMSPNet on downstream tasks such as detection, edge detection, and keypoint localization, comprehensive evaluations on segmentation tasks remain limited. In addition, this study primarily focuses on algorithm-level comparisons. Integrating advanced hardware-oriented solutions, such as novel imaging sensors or optical acquisition systems, represents another promising research direction.

Future work will focus on addressing these issues by optimizing the network components to improve their flexibility, enabling lightweight and real-time deployment. Moreover, we will expand the dataset with more challenging real-world scenarios and extend the validation to segmentation tasks.

## 5. Conclusions

This article proposes PMSPNet, a Progressive Multi-Scale Perception Network designed for the challenging task of non-uniform underwater image deblurring. PMSPNet incorporates a Hybrid Interaction Attention Module (HIAM) to effectively capture fine-grained visual textures and long-range contextual dependencies, enabling robust modeling of feature ambiguity and spatial disparity. Furthermore, the Progressive Motion-Aware Perception Branch (PMPB) is introduced to explicitly represent spatial orientation variations and progressively refine the localization of blur-affected regions. In addition, the Progressive Feature Feedback Block (PFFB) enhances feature reconstruction by leveraging multi-level information in a feedback manner, improving feature restoration quality. To enable reliable evaluation, we construct the N2UD dataset, which contains diverse non-uniform blur patterns representative of real-world underwater environments. Extensive experiments on real-world datasets validate the superiority of our method in terms of both quantitative metrics and visual quality. While achieving an inference speed of 0.01 s, it reached the highest PSNR of 25.51 dB and SSIM of 0.92 on the N2UD dataset. PMSPNet also demonstrates clear advantages in downstream tasks such as edge detection and object recognition, which enhances the visual reliability of underwater perception systems, facilitating downstream tasks such as object detection, recognition, and navigation in robotic platforms. Future efforts will focus on extending PMSPNet for real-time deployment in underwater robotic systems. Moreover, extending PMSPNet to handle real-time underwater video restoration and multimodal fusion with sonar or depth data remains a promising direction. In addition, diffusion-based generative models, despite their current limitations in computational efficiency, offer great potential for modeling complex blur patterns and realistic underwater degradations. Integrating the strengths of diffusion models into underwater deblurring frameworks will be an important avenue for our future research.

## Figures and Tables

**Table 1:** Comparison of performance on the N2UD dataset, where evaluation includes full-reference image quality metrics and resource consumption metrics. All results are reported in the format of mean ± standard deviation. ↑ indicates that a higher value is better, while ↓ indicates that a lower value is better.

Methods	PSNR ↑	SSIM ↑	FSIM ↑	LPIPS ↓	Params (M) ↓	FLOPs (G) ↓	Time (s) ↓
WFAC [25]	15.73 ± 3.21	0.66 ± 0.14	0.77 ± 0.09	0.35 ± 0.10	-	-	0.38
WWPF [57]	17.48 ± 3.59	±0.73 ± 0.13	0.82 ± 0.07	0.28 ± 0.11	-	-	0.26
HFM [58]	17.31 ± 3.18	0.76 ± 0.11	0.88 ± 0.06	0.31 ± 0.14	-	-	0.53
HLRP [59]	12.80 ± 1.97	0.22 ± 0.07	0.64 ± 0.05	0.51 ± 0.08	-	-	0.01
ACDC [60]	16.63 ± 2.90	0.70 ± 0.12	0.82 ± 0.07	0.34 ± 0.11	-	-	0.22
MMLE [61]	17.80 ± 3.73	0.73 ± 0.12	0.82 ± 0.07	0.29 ± 0.11	-	-	0.08
PCDE [62]	15.40 ± 2.96	0.62 ± 0.14	0.75 ± 0.08	0.39 ± 0.11	-	-	0.29
TEBCF [63]	17.90 ± 2.30	0.69 ± 0.12	0.80 ± 0.08	0.30 ± 0.09	-	-	1.24
CycleGAN [29]	23.91 ± 4.72	0.83 ± 0.11	0.91 ± 0.05	0.24 ± 0.01	22.76	99.364	0.03
U-Shape [49]	24.32 ± 3.87	0.83 ± 0.11	0.92 ± 0.04	0.22 ± 0.06	31.59	26.10	0.05
FUnIE-GAN [48]	21.42 ± 3.49	0.79 ± 0.09	0.90 ± 0.04	0.28 ± 0.06	3.59	26.72	0.06
Histoformer [64]	13.96 ± 2.25	0.30 ± 0.14	0.64 ± 0.07	0.72 ± 0.07	25.71	44.42	0.03
Phaseformer [65]	24.13 ± 3.17	0.64 ± 0.11	0.93 ± 0.04	0.21 ± 0.09	1.78	14.12	0.03
UIR-PolyKernel [66]	22.19 ± 4.62	0.84 ± 0.09	0.92 ± 0.04	0.25 ± 0.09	1.89	13.68	0.01
CCL-Net [67]	23.81 ± 5.39	0.83 ± 0.20	0.91 ± 0.10	0.23 ± 0.16	0.55	37.36	0.06
PUIE-Net [68]	24.40 ± 3.78	0.91 ± 0.08	0.96 ± 0.04	0.17 ± 0.08	0.83	150.69	0.13
USUIR [69]	18.79 ± 2.86	0.79 ± 0.10	0.89 ± 0.04	0.32 ± 0.08	0.23	14.88	0.01
SGUIE [70]	24.13 ± 4.50	0.87 ± 0.09	0.93 ± 0.04	0.20 ± 0.08	18.63	20.16	0.02
PMSPNet	25.51 ± 3.98	0.92 ± 0.09	0.95 ± 0.04	0.19 ± 0.08	4.44	26.77	0.01

**Table 2:** Comparison of performance on the N2UD dataset, where evaluation is based on no-reference image quality metrics. All results are reported in the format of mean ± standard deviation. ↑ indicates that a higher value is better, while ↓ indicates that a lower value is better.

Methods	UIQM ↑	UCIQE ↑	NIQE ↓	URANKER ↑	Laplacian ↑	Tenengrad ↑	Brenner ↑
WFAC [25]	3.16 ± 0.33	0.42 ± 0.02	6.02 ± 3.46	2.49 ± 0.89	0.11 ± 0.18	0.53 ± 0.23	2044.20 ± 1559.13
WWPF [57]	2.85 ± 0.40	0.44 ± 0.04	5.43 ± 2.20	2.50 ± 0.741	0.05 ± 0.08	0.44 ± 0.17	1291.93 ± 911.55
HFM [58]	2.91 ± 0.37	0.47 ± 0.03	5.49 ± 2.02	2.35 ± 0.81	0.02 ± 0.06	0.31 ± 0.14	662.62 ± 639.15
HLRP [59]	2.62 ± 0.67	0.49 ± 0.07	5.78 ± 2.25	1.69 ± 0.96	0.03 ± 0.03	0.39 ± 0.09	1016.97 ± 489.98
ACDC [60]	3.34 ± 0.20	0.38 ± 0.03	5.24 ± 1.78	2.68 ± 0.76	0.04 ± 0.08	0.42 ± 0.15	990.85 ± 790.32
MMLE [61]	2.77 ± 0.41	0.44 ± 0.04	5.56 ± 2.42	2.53 ± 0.86	0.07 ± 0.10	0.46 ± 0.19	1504.57 ± 1072.266
PCDE [62]	2.66 ± 0.68	0.46 ± 0.03	5.50 ± 1.98	2.59 ± 0.74	0.06 ± 0.08	0.50 ± 0.17	1671.21 ± 995.43
TEBCF [63]	3.00 ± 0.35	0.45 ± 0.02	5.60 ± 1.75	2.42 ± 0.69	0.049 ± 0.07	0.46 ± 0.14	1274.67 ± 699.73
CycleGAN [29]	3.19 ± 0.42	0.41 ± 0.06	4.62 ± 1.30	1.50 ± 0.81	0.01 ± 0.01	0.26 ± 0.10	448.48 ± 291.55
U-Shape [49]	3.10 ± 0.46	0.38 ± 0.05	5.05 ± 1.37	1.35 ± 0.74	0.01 ± 0.01	0.22 ± 0.09	313.03 ± 202.13
FUnIE-GAN [48]	3.10 ± 0.43	0.43 ± 0.05	4.17 ± 0.90	1.80 ± 0.78	0.02 ± 0.02	0.30 ± 0.14	607.95 ± 504.67
Histoformer [64]	3.09 ± 0.27	0.31 ± 0.04	12.00 ± 3.09	0.74 ± 0.54	0.01 ± 0.01	0.12 ± 0.04	99.17 ± 76.09
Phaseformer [65]	2.77 ± 0.39	0.43 ± 0.06	7.75 ± 6.61	1.26 ± 0.79	0.02 ± 0.03	0.24 ± 0.10	399.73 ± 353.68
UIR-PolyKernel [66]	2.91 ± 0.62	0.38 ± 0.07	5.09 ± 1.38	1.18 ± 0.99	0.01 ± 0.02	0.24 ± 0.13	435.62 ± 447.07
CCL-Net [67]	3.03 ± 0.48	0.41 ± 0.06	5.56 ± 2.05	1.46 ± 0.76	0.02 ± 0.03	0.26 ± 0.11	488.43 ± 408.52
PUIE-Net [68]	3.00 ± 0.51	0.40 ± 0.06	5.55 ± 1.85	1.42 ± 0.85	0.02 ± 0.02	0.25 ± 0.11	430.14 ± 375.36
USUIR [69]	2.96 ± 0.30	0.46 ± 0.03	4.80 ± 1.15	1.51 ± 0.82	0.01 ± 0.01	0.29 ± 0.11	528.35 ± 372.24
SGUIE [70]	2.96 ± 0.56	0.38 ± 0.07	5.46 ± 1.52	1.31 ± 0.90	0.01 ± 0.02	0.22 ± 0.11	350.12 ± 326.33
PMSPNet	3.46 ± 0.41	0.46 ± 0.06	5.30 ± 1.61	1.73 ± 0.79	0.01 ± 0.01	0.22 ± 0.09	319.80 ± 254.67

**Table 3:** Comparison of performance on the EUVP dataset, where evaluation includes full-reference and no-reference image quality metrics. All results are reported in the format of mean ± standard deviation. ↑ indicates that a higher value is better, while ↓ indicates that a lower value is better.

Methods	PSNR ↑	SSIM ↑	FSIM ↑	LPIPS ↓	UIQM ↑	UCIQE ↑	URANKER ↑
WFAC [25]	13.24 ± 2.43	0.54 ± 0.10	0.68 ± 0.07	0.45 ± 0.10	2.81 ± 0.26	0.43 ± 0.02	2.97 ± 0.90
WWPF [57]	14.62 ± 2.83	0.60 ± 0.10	0.75 ± 0.05	0.39 ± 0.09	2.68 ± 0.33	0.45 ± 0.04	2.16 ± 0.81
HFM [58]	15.13 ± 2.74	0.66 ± 0.10	0.82 ± 0.04	0.44 ± 0.11	2.93 ± 0.25	0.49 ± 0.03	2.44 ± 0.99
HLRP [59]	11.41 ± 1.86	0.17 ± 0.06	0.61 ± 0.04	0.60 ± 0.06	2.65 ± 0.59	0.50 ± 0.06	2.09 ± 0.99
ACDC [60]	14.42 ± 2.61	0.60 ± 0.10	0.75 ± 0.07	0.46 ± 0.09	3.34 ± 0.15	0.38 ± 0.03	2.98 ± 0.81
MMLE [61]	14.12 ± 2.69	0.59 ± 0.09	0.72 ± 0.06	0.41 ± 0.10	2.56 ± 0.31	0.45 ± 0.04	2.78 ± 0.98
PCDE [62]	13.55 ± 2.48	0.52 ± 0.13	0.68 ± 0.08	0.47 ± 0.11	2.37 ± 0.56	0.47 ± 0.02	2.97 ± 0.80
TEBCF [63]	17.07 ± 2.55	0.68 ± 0.09	0.79 ± 0.07	0.35 ± 0.07	2.82 ± 0.36	0.45 ± 0.03	2.59 ± 0.81
CycleGAN [29]	22.68 ± 3.52	0.79 ± 0.07	0.89 ± 0.04	0.29 ± 0.06	3.11 ± 0.50	0.40 ± 0.06	1.30 ± 0.85
U-Shape [49]	24.92 ± 3.78	0.83 ± 0.07	0.93 ± 0.02	0.23 ± 0.05	2.97 ± 0.62	0.38 ± 0.05	1.18 ± 0.84
FUnIE-GAN [48]	24.06 ± 2.60	0.79 ± 0.05	0.90 ± 0.02	0.27 ± 0.04	2.88 ± 0.57	0.41 ± 0.05	1.36 ± 0.82
Histoformer [64]	14.82 ± 2.88	0.33 ± 0.14	0.65 ± 0.07	0.71 ± 0.08	3.12 ± 0.23	0.30 ± 0.04	0.80 ± 0.50
Phaseformer [65]	23.58 ± 2.64	0.61 ± 0.07	0.91 ± 0.02	0.27 ± 0.06	2.63 ± 0.51	0.40 ± 0.05	0.98 ± 0.81
UIR-PolyKernel [66]	24.92 ± 3.86	0.87 ± 0.05	0.93 ± 0.02	0.22 ± 0.04	2.85 ± 0.74	0.40 ± 0.06	1.37 ± 0.90
CCL-Net [67]	24.55 ± 3.17	0.84 ± 0.07	0.93 ± 0.02	0.23 ± 0.04	2.97 ± 0.60	0.38 ± 0.06	1.34 ± 0.80
PUIE-Net [68]	24.71 ± 2.70	0.85 ± 0.06	0.93 ± 0.02	0.20 ± 0.04	2.97 ± 0.61	0.37 ± 0.06	1.12 ± 0.76
USUIR [69]	17.53 ± 2.47	0.73 ± 0.08	0.86 ± 0.03	0.35 ± 0.08	2.82 ± 0.22	0.47 ± 0.04	1.78 ± 0.89
SGUIE [70]	25.48 ± 3.23	0.84 ± 0.06	0.92 ± 0.03	0.24 ± 0.05	2.83 ± 0.71	0.38 ± 0.06	1.12 ± 0.87
PMSPNet	25.81 ± 3.22	0.85 ± 0.07	0.94 ± 0.02	0.21 ± 0.04	3.09 ± 0.46	0.36 ± 0.05	1.16 ± 0.78

**Table 4:** Comparison of performance on the LSUI dataset, where evaluation includes full-reference and no-reference image quality metrics. All results are reported in the format of mean ± standard deviation. ↑ indicates that a higher value is better, while ↓ indicates that a lower value is better.

Methods	PSNR ↑	SSIM ↑	FSIM ↑	LPIPS ↓	UIQM ↑	UCIQE ↑	URANKER ↑
WFAC [25]	15.35 ± 3.09	0.61 ± 0.14	0.73 ± 0.09	0.37 ± 0.08	2.76 ± 0.45	0.43 ± 0.02	2.55 ± 0.84
WWPF [57]	17.51 ± 3.44	0.70 ± 0.12	0.81 ± 0.06	0.30 ± 0.08	2.76 ± 0.41	0.45 ± 0.05	2.59 ± 0.65
HFM [58]	17.63 ± 2.93	0.74 ± 0.11	0.87 ± 0.06	0.33 ± 0.11	2.80 ± 0.31	0.47 ± 0.03	2.41 ± 0.73
HLRP [59]	13.04 ± 1.87	0.22 ± 0.08	0.64 ± 0.04	0.55 ± 0.05	2.80 ± 0.58	0.47 ± 0.09	1.65 ± 0.88
ACDC [60]	16.96 ± 2.63	0.71 ± 0.12	0.82 ± 0.06	0.33 ± 0.10	3.34 ± 0.18	0.38 ± 0.03	2.63 ± 0.72
MMLE [61]	17.59 ± 3.15	0.69 ± 0.11	0.79 ± 0.06	0.31 ± 0.08	2.55 ± 0.44	0.45 ± 0.04	2.59 ± 0.80
PCDE [62]	15.25 ± 2.30	0.59 ± 0.11	0.73 ± 0.07	0.40 ± 0.09	2.32 ± 0.57	0.47 ± 0.03	2.75 ± 0.69
TEBCF [63]	17.95 ± 2.05	0.68 ± 0.12	0.80 ± 0.08	0.31 ± 0.08	2.93 ± 0.33	0.45 ± 0.03	2.58 ± 0.64
CycleGAN [29]	24.93 ± 4.32	0.85 ± 0.11	0.92 ± 0.05	0.23 ± 0.09	3.21 ± 0.37	0.42 ± 0.06	1.63 ± 0.71
U-Shape [49]	24.94 ± 3.54	0.84 ± 0.11	0.92 ± 0.04	0.22 ± 0.07	3.10 ± 0.41	0.39 ± 0.05	1.38 ± 0.61
FUnIE-GAN [48]	21.47 ± 3.32	0.80 ± 0.10	0.90 ± 0.04	0.28 ± 0.06	3.09 ± 0.38	0.43 ± 0.05	1.84 ± 0.64
Histoformer [64]	14.05 ± 2.04	0.31 ± 0.14	0.65 ± 0.07	0.72 ± 0.07	3.07 ± 0.28	0.31 ± 0.04	0.70 ± 0.56
Phaseformer [65]	24.64 ± 3.14	0.64 ± 0.11	0.93 ± 0.04	0.20 ± 0.09	2.79 ± 0.33	0.44 ± 0.06	1.29 ± 0.70
UIR-PolyKernel [66]	22.22 ± 4.20	0.84 ± 0.09	0.92 ± 0.04	0.26 ± 0.09	2.91 ± 0.54	0.38 ± 0.07	1.16 ± 0.90
CCL-Net [67]	25.15 ± 4.45	0.87 ± 0.15	0.94 ± 0.08	0.19 ± 0.12	3.02 ± 0.43	0.41 ± 0.06	1.52 ± 0.67
PUIE-Net [68]	26.21 ± 3.63	0.90 ± 0.09	0.95 ± 0.04	0.18 ± 0.08	3.06 ± 0.44	0.39 ± 0.06	1.39 ± 0.65
USUIR [69]	18.86 ± 2.74	0.80 ± 0.11	0.89 ± 0.04	0.33 ± 0.08	2.96 ± 0.29	0.45 ± 0.04	1.44 ± 0.75
SGUIE [70]	24.59 ± 4.40	0.87 ± 0.10	0.93 ± 0.04	0.19 ± 0.08	2.96 ± 0.49	0.39 ± 0.07	1.37 ± 0.78
PMSPNet	26.46 ± 3.91	0.92 ± 0.09	0.96 ± 0.03	0.13 ± 0.07	3.31 ± 0.38	0.45 ± 0.06	1.51 ± 0.70

**Table 5:** Comparison of performance on the UIEB dataset, where evaluation includes full-reference and no-reference image quality metrics. All results are reported in the format of mean ± standard deviation. ↑ indicates that a higher value is better, while ↓ indicates that a lower value is better.

Methods	PSNR ↑	SSIM ↑	FSIM ↑	LPIPS ↓	UIQM ↑	UCIQE ↑	URANKER ↑
WFAC [25]	15.39 ± 2.25	0.66 ± 0.13	0.74 ± 0.10	0.35 ± 0.11	2.79 ± 0.52	0.43 ± 0.02	2.39 ± 0.79
WWPF [57]	17.52 ± 2.71	0.76 ± 0.10	0.84 ± 0.08	0.25 ± 0.11	2.75 ± 0.54	0.44 ± 0.04	2.54 ± 0.89
HFM [58]	17.76 ± 3.44	0.79 ± 0.11	0.89 ± 0.07	0.26 ± 0.14	2.93 ± 0.52	0.47 ± 0.03	2.33 ± 0.98
HLRP [59]	13.33 ± 1.58	0.19 ± 0.07	0.64 ± 0.04	0.55 ± 0.07	3.10 ± 0.67	0.43 ± 0.09	1.46 ± 0.88
ACDC [60]	17.70 ± 3.20	0.78 ± 0.10	0.86 ± 0.08	0.27 ± 0.12	3.39 ± 0.35	0.38 ± 0.02	2.57 ± 0.85
MMLE [61]	17.35 ± 2.91	0.73 ± 0.11	0.80 ± 0.08	0.29 ± 0.11	2.46 ± 0.57	0.45 ± 0.04	2.56 ± 0.96
PCDE [62]	15.20 ± 3.67	0.61 ± 0.19	0.75 ± 0.12	0.38 ± 0.13	2.27 ± 0.94	0.44 ± 0.02	2.69 ± 0.81
TEBCF [63]	17.68 ± 2.51	0.76 ± 0.13	0.84 ± 0.11	0.25 ± 0.10	2.84 ± 0.38	0.46 ± 0.03	2.60 ± 0.87
CycleGAN [29]	19.44 ± 4.28	0.77 ± 0.10	0.88 ± 0.06	0.27 ± 0.09	3.19 ± 0.50	0.40 ± 0.06	1.15 ± 1.02
U-Shape [49]	20.72 ± 3.59	0.81 ± 0.10	0.89 ± 0.06	0.21 ± 0.07	3.25 ± 0.43	0.37 ± 0.05	1.39 ± 1.09
FUnIE-GAN [48]	18.02 ± 2.10	0.76 ± 0.07	0.88 ± 0.04	0.29 ± 0.08	3.42 ± 0.21	0.43 ± 0.05	2.09 ± 1.06
Histoformer [64]	12.50 ± 1.62	0.23 ± 0.13	0.59 ± 0.09	0.73 ± 0.05	3.15 ± 0.22	0.32 ± 0.04	0.80 ± 0.47
Phaseformer [65]	22.41 ± 3.28	0.68 ± 0.15	0.93 ± 0.04	0.16 ± 0.09	2.82 ± 0.47	0.43 ± 0.05	1.48 ± 1.03
UIR-PolyKernel [66]	17.72 ± 4.06	0.80 ± 0.10	0.90 ± 0.05	0.24 ± 0.11	2.96 ± 0.78	0.38 ± 0.07	1.07 ± 1.44
CCL-Net [67]	16.93 ± 5.99	0.64 ± 0.31	0.79 ± 0.17	0.38 ± 0.27	3.16 ± 0.52	0.41 ± 0.06	1.32 ± 0.97
PUIE-Net [68]	22.43 ± 3.95	0.90 ± 0.07	0.94 ± 0.05	0.13 ± 0.07	3.09 ± 0.49	0.39 ± 0.07	1.45 ± 1.27
USUIR [69]	19.95 ± 3.41	0.82 ± 0.09	0.91 ± 0.06	0.24 ± 0.10	3.18 ± 0.35	0.46 ± 0.03	1.56 ± 0.98
SGUIE [70]	20.42 ± 4.47	0.86 ± 0.09	0.92 ± 0.05	0.19 ± 0.11	3.10 ± 0.63	0.37 ± 0.07	1.25 ± 1.35
PMSPNet	22.43 ± 3.37	0.86 ± 0.08	0.92 ± 0.05	0.21 ± 0.10	3.27 ± 0.39	0.39 ± 0.05	1.53 ± 1.08

**Table 6:** Comparison of performance using different components, where evaluation includes full-reference and no-reference image quality metrics. All results are reported in the format of mean ± standard deviation. ↑ indicates that a higher value is better. ✓ indicates the model is evaluated w/the corresponding module, while ✗ denotes the model is evaluated w/o the corresponding module.

ButterWorth	Deformable	PMPB	PFFB	PSNR ↑	SSIM ↑	FSIM ↑	UIQM ↑	UCIQE ↑
✗	✓	✓	✓	25.98 ± 4.20	0.88 ± 0.09	0.94 ± 0.04	3.08 ± 0.40	0.39 ± 0.06
✓	✗	✓	✓	17.18 ± 5.14	0.73 ± 0.13	0.87 ± 0.06	2.31 ± 0.84	0.41 ± 0.08
✗	✗	✗	✓	25.99 ± 4.29	0.89 ± 0.09	0.94 ± 0.04	3.09 ± 0.39	0.40 ± 0.06
✓	✓	✓	✗	24.78 ± 4.07	0.87 ± 0.09	0.94 ± 0.04	3.08 ± 0.41	0.39 ± 0.06
✓	✓	✓	✓	25.51 ± 3.98	0.92 ± 0.09	0.95 ± 0.04	3.46 ± 0.41	0.46 ± 0.06

**Table 7:** Comparison of performance using different loss functions, where evaluation includes full-reference and no-reference image quality metrics. All results are reported in the format of mean ± standard deviation. ↑ indicates that a higher value is better. ✓ indicates the model is evaluated w/the corresponding module, while ✗ denotes the model is evaluated w/o the corresponding module.

Charbonnier	FFT	LAB	LCH	VGG	Color	PSNR ↑	SSIM ↑	FSIM ↑	UIQM ↑	UCIQE ↑
✓	✗	✗	✗	✗	✗	24.00 ± 3.98	0.86 ± 0.09	0.91 ± 0.04	2.90 ± 0.45	0.41 ± 0.07
✓	✗	✗	✗	✗	✓	21.27 ± 4.31	0.83 ± 0.09	0.91 ± 0.04	2.95 ± 0.41	0.35 ± 0.06
✓	✗	✗	✗	✓	✓	21.43 ± 4.21	0.83 ± 0.09	0.91 ± 0.04	3.08 ± 0.37	0.35 ± 0.06
✓	✗	✗	✓	✓	✓	22.53 ± 4.36	0.84 ± 0.09	0.92 ± 0.04	3.08 ± 0.37	0.37 ± 0.06
✓	✗	✓	✓	✓	✓	24.06 ± 3.84	0.85 ± 0.09	0.92 ± 0.04	3.08 ± 0.38	0.39 ± 0.06
✓	✓	✓	✓	✓	✓	25.51 ± 3.80	0.92 ± 0.09	0.95 ± 0.04	3.46 ± 0.41	0.46 ± 0.06

## Data Availability

Publicly available datasets were analyzed in this study. These data can be found at https://github.com/UI2025/N2UD (N2UD), accessed on 28 August 2025; https://irvlab.cs.umn.edu/resources/euvp-dataset (EUVP), accessed on 17 May 2025; https://lintaopeng.github.io/_pages/UIE%20Project%20Page.html (LSUI), accessed on 17 May 2025; https://li-chongyi.github.io/proj_benchmark.html (UIEB), accessed on 17 May 2025; and https://github.com/chongweiliu/DUO (DUO), accessed on 18 May 2025.
